# Modulating the skin mycobiome-bacteriome and treating seborrheic dermatitis with a probiotic-enriched oily suspension

**DOI:** 10.1038/s41598-024-53016-0

**Published:** 2024-02-01

**Authors:** Mauro Truglio, Francesca Sivori, Ilaria Cavallo, Elva Abril, Valerio Licursi, Giorgia Fabrizio, Giorgia Cardinali, Marco Pignatti, Luigi Toma, Floriana Valensise, Antonio Cristaudo, Fulvia Pimpinelli, Enea Gino Di Domenico

**Affiliations:** 1 Microbiology and Virology, San Gallicano Dermatological Institute, IRCCS, 00144 Rome, Italy; 2 Institute of Molecular Biology and Pathology, National Research Council of Italy, 00185 Rome, Italy; 3 Department of Biology and Biotechnology C. Darwin, Sapienza University of Rome, 00185 Rome, Italy; 4 Cutaneous Physiopathology, San Gallicano Dermatological Institute, IRCCS, 00144 Rome, Italy; 5 Clinica Tarabini, 41012 Carpi, Italy; 6 Medical Directorate, IRCCS Regina Elena National Cancer Institute, 00144 Rome, Italy; 7 Clinical Dermatology, San Gallicano Dermatological Institute, IRCCS, 00144 Rome, Italy

**Keywords:** Microbiology, Diseases, Health care, Signs and symptoms

## Abstract

Seborrheic dermatitis (SD) affects 2–5% of the global population, with imbalances in the skin microbiome implicated in its development. This study assessed the impact of an oily suspension containing *Lactobacillus crispatus* P17631 and *Lacticaseibacillus paracasei* I1688 (termed EUTOPLAC) on SD symptoms and the skin mycobiome-bacteriome modulation. 25 SD patients were treated with EUTOPLAC for a week. Symptom severity and skin mycobiome-bacteriome changes were measured at the start of the treatment (T0), after seven days (T8), and three weeks post-treatment (T28). Results indicated symptom improvement post-EUTOPLAC, with notable reductions in the *Malassezia* genus. Concurrently, bacterial shifts were observed, including a decrease in *Staphylococcus* and an increase in *Lactobacillus* and *Lacticaseibacillus*. Network analysis highlighted post-EUTOPLAC instability in fungal and bacterial interactions, with increased negative correlations between *Malassezia* and *Lactobacillus* and *Lacticaseibacillus* genera. The study suggests EUTOPLAC's potential as a targeted SD treatment, reducing symptoms and modulating the mycobiome-bacteriome composition.

## Introduction

Seborrheic dermatitis (SD) is a prevalent, chronic inflammatory skin disorder affecting approximately 2–5% of the population worldwide. SD is characterized by thin patches covered with oily scales, primarily occurring in areas with a high concentration of sebaceous glands, including the scalp, face, chest, back, and body folds^1,2^.

The pathogenesis of SD is multifactorial, involving genetic predisposition, immune system dysregulation, and environmental factors^3^. Moreover, the skin microbiome, particularly the complex interactions between commensal fungi and bacteria, is thought to play a crucial role in SD development and progression^4^. Disruption of the skin microbiome, known as dysbiosis, has been implicated in various skin disorders, including SD, atopic dermatitis, acne, and psoriasis^5,6^.

*Malassezia* spp., a group of lipophilic yeasts, is considered the main fungi involved in SD pathogenesis, triggering an immune response that results in inflammation and scaling^7^. The presence of *Malassezia* spp. has been shown to play a major role in the development of SD and an increased *M. restricta*/*M. globosa* ratio was associated with SD of the scalp^8^. Although *Malassezia* spp. is considered a natural component of the human skin microbiota, under certain conditions, they can overgrow and lead to an inflammatory response^9^. Indeed, *Malassezia* interacts with the skin surface either directly through the activation of the aryl hydrocarbon receptor or via their metabolites, causing a disruption in the skin barrier and initiating an inflammatory response^10^. Different studies exploring the skin microbial composition have shown that other microorganisms, such as *Staphylococcus*, *Candida*, *Aspergillus*, and *Filobasidium*, are overrepresented in individuals with SD/dandruff^11–17^. Microbial communities on the scalp and hair surfaces of individuals with dandruff appear more unstable than those in healthy individuals, making these networks possibly more vulnerable to environmental changes. Notably, *Lactobacillus* bacteria demonstrate numerous interactions within the microbial community on the scalp and hair surfaces, suggesting that targeting *Lactobacillus* could be a strategic approach for enhancing scalp health^18^. Recent studies have highlighted the potential of probiotics, including *Lactobacillus* genera, in modulating the skin microbiome and alleviating symptoms of various skin conditions, including atopic dermatitis and acne^19^. Probiotics are known to modulate the immune system, inhibit the growth of pathogenic microorganisms, and maintain the balance of the skin microbiome^20^. However, little information and clinical studies analyzing the efficacy of topically applied probiotic products are available^19^.

This study investigates the modulatory effects of a topical oily suspension containing the probiotic bacteria *Lactobacillus crispatus* P17631 and *Lacticaseibacillus paracasei* I1688 (EUTOPLAC) on the skin mycobiome-bacteriome composition and its potential in alleviating SD severity.

## Results

### Baseline demographics and the SD area severity index (SDASI) score

A total of 25 patients were enrolled in the study. Baseline characteristics are reported in Table 1. Most patients (60.0%) had severe SD, as assessed by the SDASI score.

**Table 1:** Baseline demographics and disease characteristics.

Patient characteristics	
Patients (N)	25
Age (years)	32 (23–63)
Male/Female (%)	56/44
SDASI* (%)	
Moderate	40.0
Severe	60.0

Age is expressed as median (range).

*SDASI seborrheic dermatitis area severity index.

**Figure 1:**
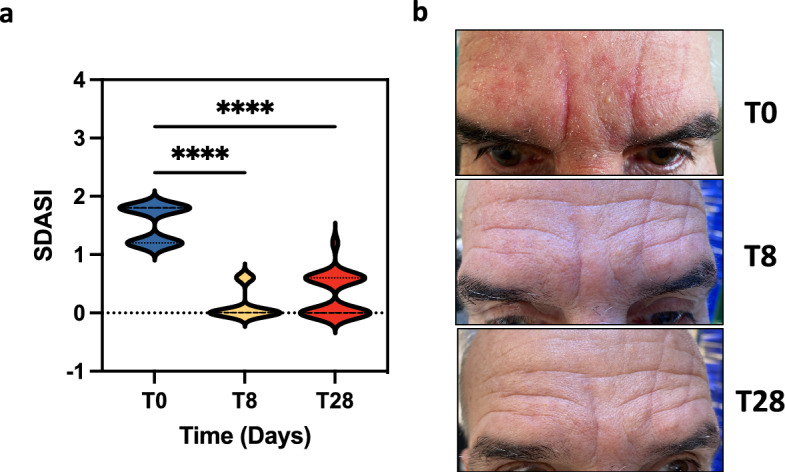
Clinical parameters in SD patients treated with EUTOPLAC. (**a**) The severity of erythema, desquamation, and pruritus in the forehead and nasolabial regions was evaluated according to the Seborrheic Dermatitis Area Severity Index (SDASI). Assessments were made at three distinct time points: before treatment (T0), one-week post-treatment (T8), and three weeks post-treatment (T28). Statistical differences were determined using the Friedman teste and post hoc Nemenyi test. (**b**) Images showing the skin lesions on a patient's face with seborrheic dermatitis at different time points.

In this study, all SD patients received EUTOPLAC treatment for one week. Baseline SDASI scores were significantly higher compared to scores from patient’s post-treatment at both the one-week (T8; *P* < 0.0001) and three-week (T28; *P* < 0.0001) time points. Importantly, no significant changes in SDASI scores were observed between the one-week post-treatment (T8) and the three-week post-treatment (T28) time points (Fig. 1a, b).

### Skin surface mycobiome of SD patients treated with EUTOPLAC

**Figure 2:**
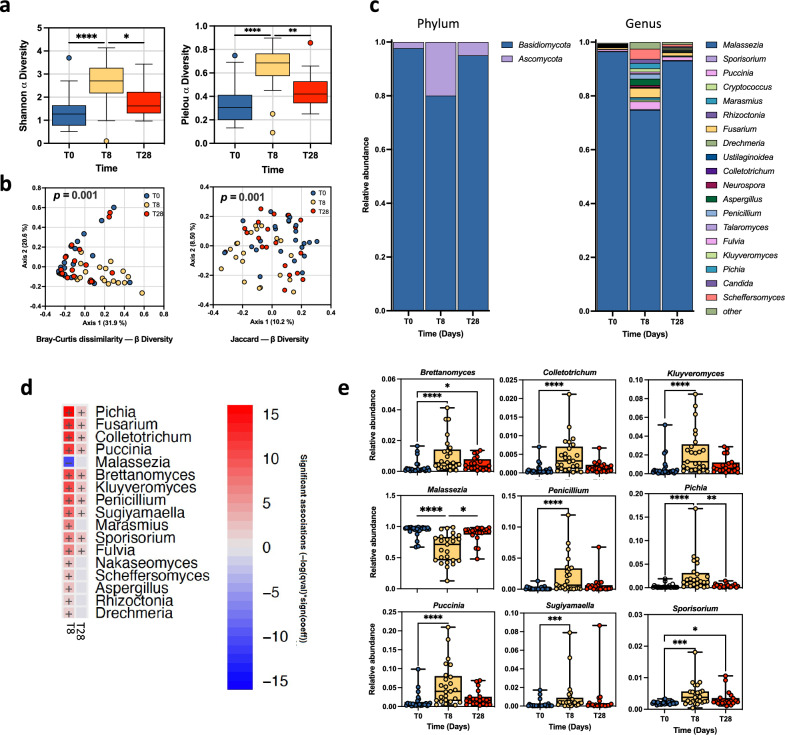
Mycobiome taxonomic composition and diversity. (**a**) Boxplot showed the fungal diversity in SD patients at T0, T8, and T28. Alpha diversity was calculated using the Shannon diversity index and Pielou Evenness index at the genus level. Statistical differences were determined using the Kruskal–Wallis test. (**b**) Bray Curtis and Jaccard beta diversity was calculated at the genus level and represented as principal coordinate analysis (PCoA). PERMANOVA test was used to assess significance. (**c**) The bar plot depicted the fungal mean relative abundance at the phylum and genus levels. (**d**) Microbiome Multivariable Associations with Linear Models (MaAsLin 2) heatmap showing significant variations in bacterial abundance at the genus level at T8 and T28 compared to T0. The color scale bar shows positive (red) and negative (blue) correlations between fungal genera, ranging from the highest positive normalization (+ 15) to the lowest one (− 15). **e** Relative abundance for the indicated fungal genera at T0, T8, and T28. Significance was assessed by the Kruskal Wallis static test. *, *P* < 0.05; **, *P* < 0.01; ***, *P* < 0.001, ****, *P* < 0.0001.

A total of 989,065 ITS2 16 S rRNA filtered reads were acquired, with an average sequencing depth of 14,129 reads/sample for mycobiome, respectively. Alpha diversity of the skin from SD patients was analyzed at the time of enrollment (T0), after seven days of treatment (T8), and three weeks post-treatment (T28) using the Shannon diversity index and Pielou evenness index. The mycobiome showed a significant (*P* < 0.0001) increase in the Shannon index at T8 (Mean ± SD = 2.66 ± 0.99) compared to T0 (Mean ± SD = 1.37 ± 0.07) and T28 (Mean ± SD = 1.84 ± 0.67) (Fig. 2a). Similarly, a significant (*P* < 0.0001) increase in the Pielou index was observed in T8 (Mean ± SD = 0.65 ± 0.18) samples than in T0 (Mean ± SD, 0.33 ± 0.16) and T28 (Mean ± SD, 0.45 ± 0.15) (Fig. 2a). Bray Curtis and Jaccard beta diversity, represented as principal coordinate analysis (PCoA), supported previous findings showing a distinctive spatial clusterization of T8 samples compared to T0 and T28 (Fig. 2b). Our results showed that the composition and diversity of fungi (Fig. 2a, b) were significantly altered at T8 compared to T0 and T28 sites. Moreover, there were no significant changes in both α-diversity and β-diversity between T0 and T28 in fungi (Fig. 2a, b).

The dominant fungal phyla in the skin of SD subjects were Basidiomycota and Ascomycota (Fig. 2c). Notably, the relative abundance of Basidiomycota was 97.7% at T0, 80.0% at T8, and 95% at T28 (Fig. 2c). *Malassezia* was the most abundant fungal genus in every group studied, albeit with certain fluctuations across different time points. Specifically, the proportion of *Malassezia* in the fungal mycobiome was 96.5% at T0, 74.8% at T8, and 93.0% at T28 (Fig. 2c).

Comparative analysis revealed significant differences in fungal abundance at the genus level (Fig. 2c). Notably, *Malassezia* significantly decreased at T8 compared to T0 (*P* < 0.0001) and T28 (*P* = 0.0124) (Fig. 2d). Accordingly, the decrease of the *Malassezia* at T8 was accompanied by a significant increase in most of the other fungal genera analyzed (Fig. 2d, e). In particular, at T8, a significant inverse correlation was observed between the *Malassezia* genus and 18 over the 32 (56%) fungal genera of the skin mycobiome of SD patients (Supplementary Fig. 1).

### EUTOPLAC does not impact the skin bacteriome diversity

**Figure 3:**
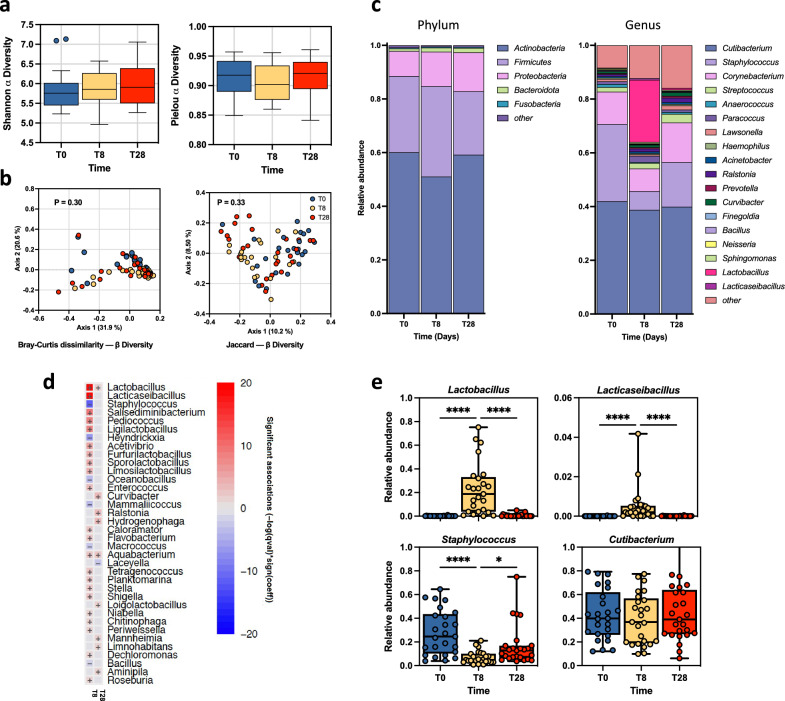
Bacteriome taxonomic composition and diversity. (**a**) Boxplot showed the bacterial diversity in SD patients at T0, T8, and T28. Alpha diversity was calculated using the Shannon diversity index and Pielou Evenness index at the genus level. Statistical differences were determined using the Kruskal–Wallis test. (**b**) Bray Curtis and Jaccard beta diversity was calculated at the genus level and represented as principal coordinate analysis (PCoA). PERMANOVA test was used to assess significance. (**c**) The bar plot depicted the bacterial mean relative abundance at the phylum and genus levels. (**d**) Microbiome Multivariable Associations with Linear Models (MaAsLin 2) heatmap showing significant variations in bacterial abundance at the genus level at T8 and T28 compared to T0. The color scale bar shows positive (red) and negative (blue) correlations between bacterial genera, ranging from the highest positive normalization (+ 20) to the lowest one (− 20). (**e**) Relative abundances for the indicated bacterial genera at T0, T8, and T28. Significance was assessed by the Kruskal Wallis static test. *, *P* < 0.05; **, *P* < 0.01; ***, *P* < 0.001, ****, *P* < 0.0001.

A total of 864,926 filtered 16S rRNA reads were acquired for the bacteriome analysis, with an average sequencing depth of 11,532 reads per sample. Analysis of the bacteriome composition revealed that alpha and beta diversity indices remained consistent across all samples, with no statistically significant differences identified between the T0, T8, and T28 time points (Fig. 3a, b). At the phylum level, skin samples were dominated by Actinobacteria, Firmicutes, Proteobacteria (Fig. 3c). At T0, the bacteriome of SD patients was primarily characterized by *Cutibacterium* (41.954%), *Staphylococcu*s (28.715%), *Corynebacterium* (12%), and *Streptococcus* (1.2%) genera (Fig. 3c). Interestingly, *Lactobacillus* and *Lacticaseibacillus*, the two probiotics in the EUTOPLAC formulation, represented only 0.127% and 0.003% of the sequences, respectively. After EUTOPLAC administration (T8), the relative abundance of the *Lactobacillus* and *Lacticaseibacillus* genera showed a marked increase to 23.129% and 0.573%, respectively. Notably, among the two dominant genera, a targeted reduction was observed in *Staphylococcus* (6.917%) at T8, while the abundance of *Cutibacterium* (38.761%) remained unaffected. At the T28, the relative abundance of *Cutibacterium* (39.909%), *Staphylococcus* (16.598%), *Lactobacillus* (0.633%), and *Lacticaseibacillus* (0.006%) genera had returned to levels comparable to those reported at T0.

The impact of EUTOPLAC application on bacterial signatures was further investigated using MaAsLin2 (Fig. 3d). Notably, a significant increase was observed in the relative abundance of the *Lactobacillus* and *Lacticaseibacillus* genera at the T8 time point compared to T0 (*P* < 0.0001) and T28 (*P* < 0.0001), as reported in Fig. 3d. Conversely, the relative abundance of the *Staphylococcu*s genus showed a decrease at T8, compared to T0 (*P* < 0.0001) and T28 (*P* = 0.0115). Finally, the relative abundance of the *Cutibacterium* genus remained consistent across all time points (Fig. 3e).

**Figure 4:**
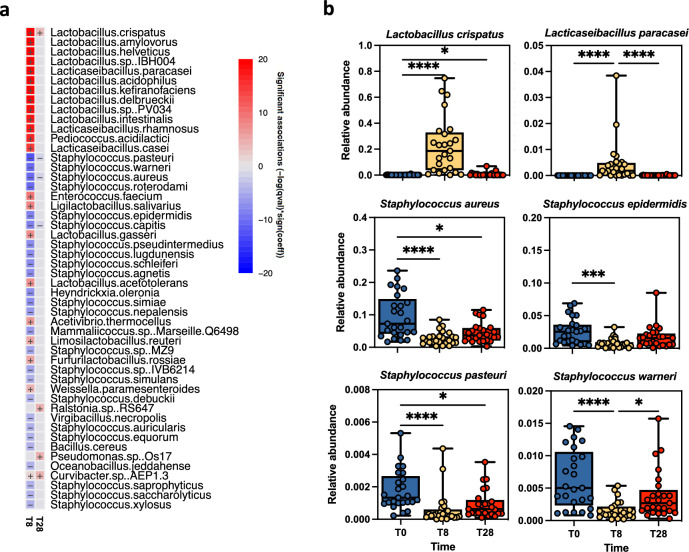
Bacteriome signature of SD patients at the species level. (**a**) Microbiome Multivariable Associations with Linear Models (MaAsLin 2) heatmap showing significant variations in bacterial abundance at the species level across different time points in SD patients. Cells denoting significant associations are colored (red or blue) with a plus ( +) or minus ( −) sign indicating the direction of the association. (**b**) Boxplots showing the relative abundances of the indicated bacterial species across time points. Significance was assessed by the Kruskal Wallis static test. *, *P* < 0.05; **, *P* < 0.01; ***, *P* < 0.001, ****, *P* < 0.0001.

The subsequent analysis at the species level revealed a significant increase for both *L. crispatus* and *L. paracasei* (*P* < 0.0001) and different *Lactobacillus* species at T8 compared to T0 and T28 (Fig. 4a, b). The relative abundances of most *Staphylococcus* species significantly varied across the time points (Fig. 4b). In particular, a significant reduction was observed at T8 compared to T0 in the relative abundance of *S. aureus* (*P* < 0.0001) and *S. epidermidis* (*P* = 0.0001), which were two of the most represented *Staphylococcus* species in the skin of SD patients. Moreover, a reduction in *S. aureus* was also observed at T28, while at the same time, the relative abundance of *S. epidermidis* returned comparable to T0. These findings imply that the weekly application of EUTOPLAC caused a temporal modulation of the bacteriome composition at the genus and species level in the skin of SD patients.

### Co‑occurrence/co‑exclusion network analysis on fungi and bacteria of SD patients treated with EUTOPLAC

**Figure 5:**
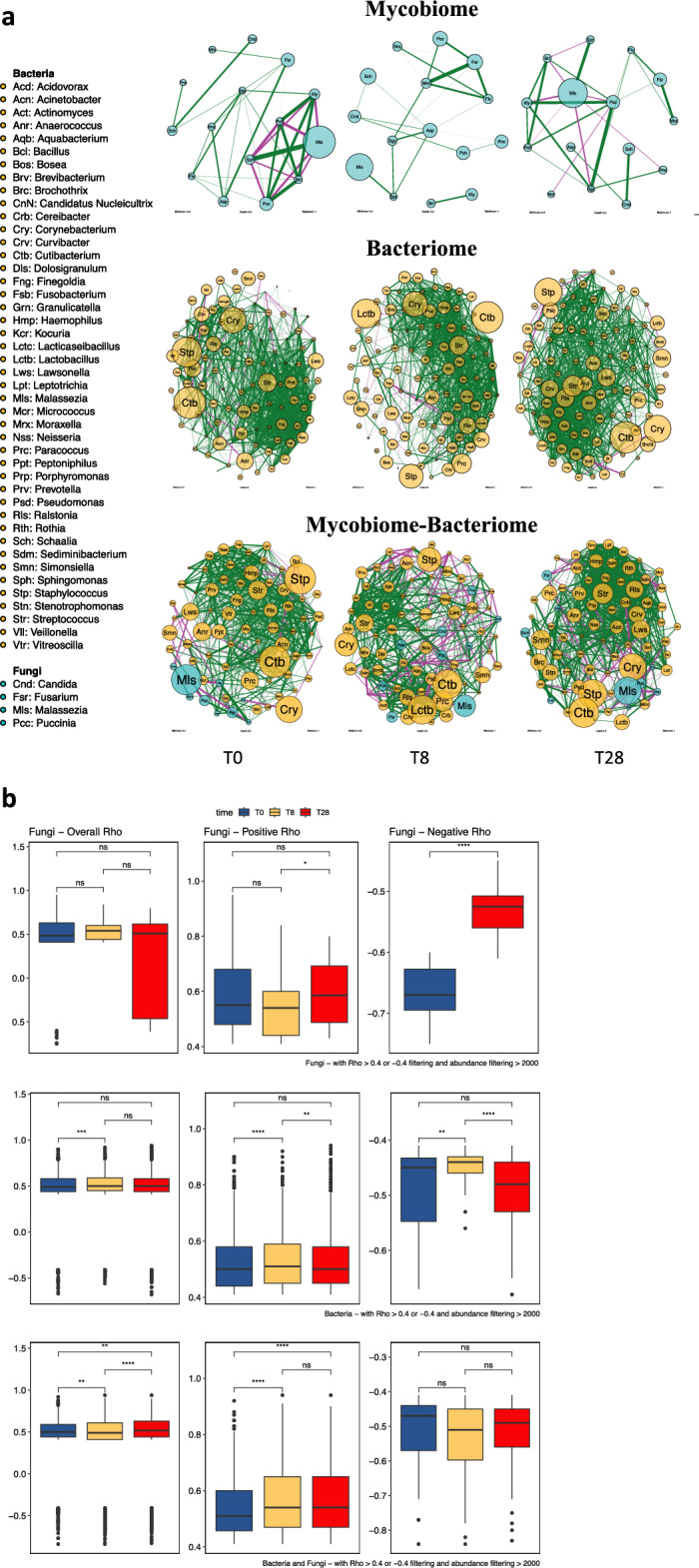
Skin mycobiome-bacteriome networks. (**a**), Fungal and bacterial co-occurrence and co-exclusion network attributes at T0, T8, and T28. Node size reflects the log-transformed relative abundance level of the mycobiome-bacteriome. The thickness of the edges corresponds to the | r | value of the Spearman correlation coefficient. The color of the edges corresponds to the positive (> 0.4) (green) or negative (< -0.4) (purple) relationship. Edge length is not indicative of any particular attribute. (**b**), Differences in Spearman’s r values between T0, T8, and T28 (*, *P* < 0.05; **, *P* < 0.01; ***, *P* < 0.001, ****, *P* < 0.0001; ns, not significant) determined via the Wilcoxon test.

The network analysis of the mycobiome (Fig. 5) indicated that positive correlations (Spearman’s r > 0) (Fig. 5b) remained consistent across T0, T8, and T28, with a significant increase from T8 to T28 (*P* = 0.028). In contrast, negative correlations (r < 0) were more abundant at T0 compared to T28 (*P* < 0.0001), with *Malassezia* being predominantly involved in these negative interactions. Interestingly, the fungal community at T8 displayed no negative correlations.

The overall bacterial correlation was significantly lower at T0 than at T8 (*P* = 0.0003). At T8, all positive correlations (r > 0) were higher compared to T0 (*P* < 0.0001) and T28 (*P* = 0.005). Conversely, negative correlations (r < 0) were lower at T8 compared to T0 (*P* = 0.006) and T28 (*P* < 0.0001). The increase in competition creates negative feedback loops that have a stabilizing effect. Similarly, reduced positive feedback improves stability^18,21,22^. These findings suggest that the bacterial community at T8 was more unstable than those established at T0 and T28 as a result of EUTOPLAC application.

The overall correlation between fungal and bacterial networks (Fig. 5) was lower at T8 compared to both T0 (*P* = 0.006) and T28 (*P* < 0.0001). All positive correlations (r > 0) were lower at T0 compared to T8 (*P* < 0.0001) and T28 (*P* < 0.0001). Meanwhile, all negative correlations (r < 0) remained consistent across T0, T8, and T28. The increase of positive feedback between fungi and bacteria observed at T8 and T28 may represent a parameter associated with the instability of the microbial community network after EUTOPLAC application.

**Figure 6:**
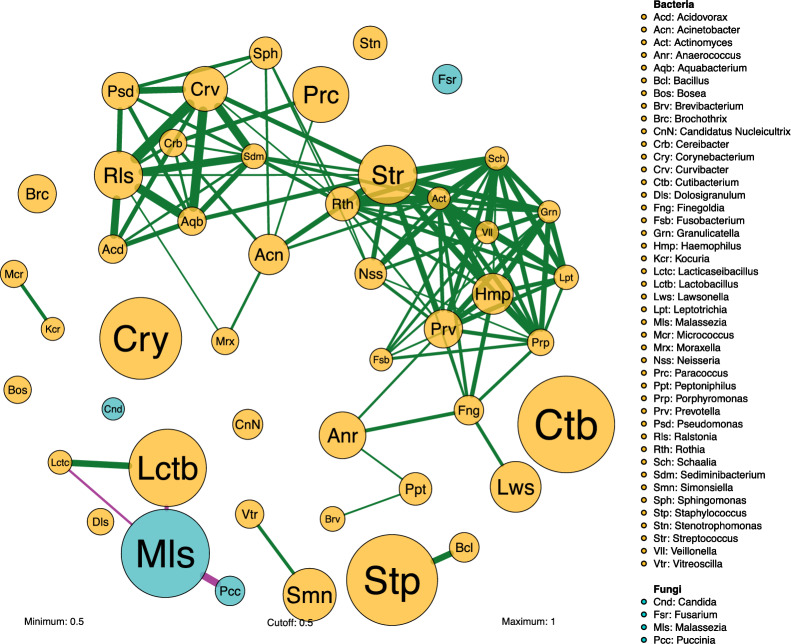
Cumulative skin mycobiome-bacteriome networks with high abundance. Each node shows one taxon of fungi (blue) or bacteria (yellow) with an average abundance greater than 4000 reads in all samples analyzed. Node size reflects the log-transformed relative abundance level of the mycobiome-bacteriome. The thickness of the edges corresponds to the | r | value of the Spearman correlation coefficient. The color of the edges corresponds to the positive (> 0.5) (green) or negative (< −0.5) (purple) relationship. Edge length is not indicative of any particular attribute.

Finally, to identify the network of highly abundant microbial genera, the relationships of | r |> 0.5 and average abundance greater than 4000 reads at T0, T8, and T28 were used to filter and reconstruct the network (Fig. 6). Most of the inter-domain associations detected were positive. Some of the strongest positive correlations were observed between a wide range of bacteria. In contrast, negative correlations all involved *Malassezia.* Specifically, the most abundant fungal genus, *Malassezia*, was negatively related to *Lactobacillus* and *Lacticaseibacillus* genus. In turn, *Lactobacillus* and *Lacticaseibacillus* exhibited a positive correlation with each other. Notably, the three dominant bacterial genera, *Cutibacterium*, *Staphylococcus*, and *Corynebacterium*, had minimal to no interactions with the broader microbial community, suggesting a central role in maintaining microbial community stability.

### Lactobacillus crispatus P17631 and Lacticaseibacillus paracasei I1688 adhesion and biofilm formation

**Figure 7:**
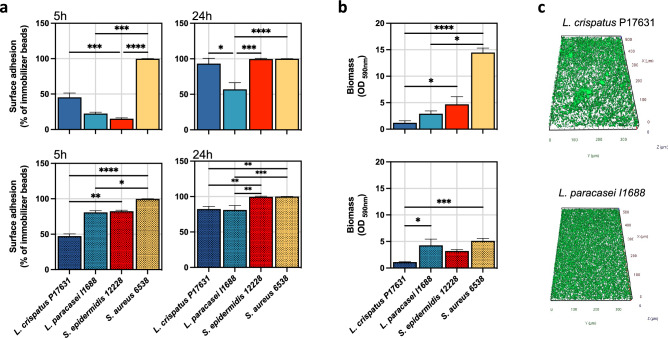
Surface adhesion and biofilm formation in *Lactobacillus crispatus* P17631 and *Lacticaseibacillus paracasei* I1688 strains. Experiments were conducted under aerobic (solid bars) and anaerobic (filled bars) conditions. *S. epidermidis* 12,228 and *S. aureus* 6538 from the American Type Culture Collection (ATCC) were used as reference strains. (**a**), The level of bacterial surface adhesion, quantified using the BioFilm Ring Test at 5 and 24 h. (**b**), The quantity of biofilm biomass produced after 48 h as measured by crystal violet assays (optical density (OD) at 590 nm). (**c**) Reconstructed three-dimensional (3D) images of biofilm formation of *Lactobacillus crispatus* P17631 and *Lacticaseibacillus paracasei* I1688 strains after 48 h of incubation in BHI at 37 °C.The graphs represent the mean values and standard errors derived from three independent experiments of duplicate samples. Significance was assessed by using the Kruskal Wallis static test. *, *P* < 0.05; **, *P* < 0.01; ***, *P* < 0.001, ****, *P* < 0.0001.

Surface adhesion and biofilm formation are central to bacterial persistence on the skin surface^23^. Given their significance, the capacity of *L. crispatus* P17631 and *L. paracasei* I1688 to adhere to surfaces and form biofilms was investigated using the BRT (Fig. 7a). Bacterial adhesion at 5 and 24 h was measured in aerobic and anaerobic growth conditions. *S. aureus* 6538 and *S. epidermidis* 12,228 were used as controls. Interestingly, *L. paracasei* I1688 displayed lower surface adhesion (*P* = 0.012) under aerobic conditions after 5 and 24 h than *L. crispatus* P17631. In contrast, under anaerobic conditions, no significant difference was observed between *L. crispatus* P17631 and *L. paracasei* I1688 regarding surface adhesion at both time points (Fig. 7a). Biofilm formation was quantified using crystal violet (CV) to assess biomass 48 h post-incubation (Fig. 7b) and fluorescence microscopy analysis (Fig. 7c). *L. crispatus* P17631 and *L. paracasei* I1688 exhibited similar biomass levels in aerobic conditions. However, under anaerobic conditions, *L. paracasei* I1688 produced more biomass (*P* = 0.015) than *L. crispatus* P17631. These findings suggest that growth conditions significantly impact bacterial growth, and *L. crispatus* P17631 and *L. paracasei* I1688 perform differently in surface adhesion and biomass production. Specifically, *L. crispatus* P17631 outperformed *L. paracasei* I1688 in surface adhesion under aerobic conditions after 5 and 24 h. However, this advantage was not observed under anaerobic conditions. Conversely, *L. paracasei* I1688 produced more biomass than *L. crispatus* P17631 under aerobic and anaerobic conditions after 48 h of incubation.

## Conclusion

SD is a chronic skin condition that affects up to approximately five percent of the general population. Although the exact pathogenesis of SD is still not fully understood, recent studies have highlighted the role of the skin microbiome in the development and progression of the disease^11,12^. In this study, we examined the impact of EUTOPLAC administration on the severity of SD symptoms. The results demonstrate a significant reduction in the SDASI score following treatment, with notable clinical improvement persisting from one week (T8) to three weeks post-treatment (T28). This finding highlights the therapeutic effectiveness of EUTOPLAC in alleviating SD symptoms and offers good insights into new treatment modalities based on probiotics. Various methods, including antifungals and topical corticosteroids, have been proposed to manage SD. However, the potential activity of topical probiotics in treating SD is still largely unexplored, underscoring the importance and novelty of our findings^24^. In a double-blind study involving 60 SD patients, superficial *Vitreoscilla filiformis* preparation decreased symptoms such as itching, erythema, and scaling. On a cellular level, this bacterial lysate boosted the activity of skin dendritic cells' IL-10 and increased regulatory T lymphocyte activity^25^. Another study showed that oral administration of *L. paracasei* NCC2461 ST11 significantly reduced symptoms, accompanied at the subcellular level by an increase in IL-10 production^26^. Besides, *L. paracasei* showed positive effects in lowering dandruff by increasing hydration and restoring the barrier function of the scalp^27^.

This report showed a pronounced shift in the mycobiome composition following EUTOPLAC treatment, which aligns with recent studies illustrating the dynamic nature of skin mycobiome in response to external factors^28^. The significant increase in the Shannon and Pielou diversity indices at T8 indicates a transient modulation in fungal diversity immediately post-treatment. The dominant fungal phylum, Basidiomycota, and the *Malassezia* genus exhibited a noticeable decrease at T8. However, by T28, Basidiomycota and *Malassezia* relative abundances had rebounded towards baseline levels. These observations suggest a transient and targeted alteration in fungal composition and diversity due to EUTOPLAC treatment. Previous research has implicated the hyperactivity of sebaceous glands coupled with the concurrent proliferation of *Malassezia* species as fundamental factors triggering SD^29^. Indeed, the excessive presence of various *Malassezia* species, particularly in seborrheic regions, may play a key role in exacerbating the condition. Furthermore, the noticeable benefits derived from antifungal treatments in managing this skin condition underscore the critical role of *Malassezia* in its pathogenesis. Thus, targeted therapies that regulate microbial balance may represent an effective approach to treating this dermatological condition^30^. Current understanding posits that SD results from the skin's inflammatory response to the free fatty acids produced by *M. furfur*^31,32^. Furthermore, *M. furfur* can generate metabolites that stimulate the aryl hydrocarbon receptor, potentially influencing the function of antigen-presenting cells^33^. In this study, we decided, for the fungal taxa, to limit our analysis to the genus level. Indeed, short amplicon sequences provide limited resolution for many fungi, which is insufficient for species-level characterization^34,35^. This limitation is particularly relevant for fungal species for which reliable ITS sequences are not yet available. This lack of reference data limits the accuracy of species-level identification. Furthermore, the quality of available ITS sequences in these databases varies, introducing additional errors in species-level identification. These challenges underscore the complexity of the fungal kingdom and the need for continued development of analytical tools and databases to improve the accuracy of species-level identification in mycobiome studies.

A previous investigation suggested that, alongside *Malassezia*, alterations in the bacterial microbial communities may also play a role in SD pathogenesis^12^.

Our study demonstrated that alpha and beta diversity indices and the dominant bacterial phyla and genera showed no statistically significant alterations. In contrast to the mycobiome findings, the bacterial community remained remarkably stable across all time points, echoing previous findings on the resilience of the skin bacteriome under various treatments^36^. Yet, a more in-depth analysis revealed interesting dynamics. In particular, the relative abundances of *Lactobacillus* and *Lacticaseibacillus*, the two probiotic genera present in EUTOPLAC, increased substantially at T8. This was accompanied by a notable reduction in the genus *Staphylococcus*. By T28, these fluctuations had largely reverted, suggesting that the impact of EUTOPLAC on the bacterial community is temporal and does not result in long-term shifts in the bacteriome composition. Based on previous studies, microbial communities that revert to their initial structure after perturbation are considered stable. In this context, the conditions that shape the microbial community in subjects with SD appear to be instrumental in modulating the transient response following EUTOPLAC application^21^.

The genus *Cutibacterium* remained unaffected throughout, indicating EUTOPLAC’s specific impact on the *Staphylococcus* genus. Interestingly, *Staphylococcus* and *Cutibacterium*, the two prevalent yet mutually inhibitory bacterial genera found on the scalp, seem to affect dandruff primarily through their balance^37^. The relative abundances of two prominent *Staphylococcus* species, *S. aureus* and *S. epidermidis*, significantly decreased at T8. Interestingly, while *S. epidermidis* levels recovered by T28, *S. aureus* relative abundance remained significantly low compared to T0, suggesting a more sustained impact on this pathogen in SD. This finding resonates with previous works hinting that a possible contribution of *S. aureus* in SD pathogenesis^24^. In particular, the relative abundance of *S. aureus* was found to be significantly higher in patients with SD as reported for the healthy control subjects^12,38^. Species-level analysis post-EUTOPLAC treatment also revealed a significant uptick in *L. crispatus* and *L. paracasei* at T8, which is not sustained at T28. The high prevalence of *L. crispatus* correlates with its 29:1 ratio over *L. paracasei* in the EUTOPLAC formulation, suggesting that the administered ratio heavily influences colonization patterns. The findings emphasize the potential of leveraging formulation ratios to optimize probiotic efficacy for skin health, warranting further investigation into the mechanisms driving these species-specific colonization trends.

Network analysis showed that the mycobiome displayed distinct temporal patterns of co-occurrence and co-exclusion. Notably, the consistent positive correlations across T0, T8, and T28, particularly the surge from T8 to T28, are evocative of mutualistic associations among certain fungi during these time points^39^. Moreover, *Malassezia*'s predominant involvement in negative correlations, especially at T0, suggests its potential role in competing with other fungi and in shaping the community structure^18^. Negative interactions can stabilize microbial communities by preventing genus dominance. This can lead to increased resilience against disturbances. In contrast, positive feedback loops contribute to instability by amplifying disturbances within the population^21^. The absence of negative correlations at T8 is puzzling and necessitates further exploration. Since stability is associated with competition, the absence of negative interactions may represent a sign of instability of the fungal community resulting from the EUTOPLAC application.

Similarly, in the bacterial population, an increase in positive interactions coupled with a significant reduction in negative feedback loops at T8 underscores the notion of a shift of the community from its original equilibrium or more stable condition^21,22^.

Lastly, the lower overall correlation between fungal and bacterial networks at T8 further reinforces the idea of a disturbance in these communities^18^. Notably, the rise in positive feedback between fungal and bacterial communities after EUTOPLAC application might perturb stability, requiring further mechanistic exploration.

The filtering approach highlighted the core microbial network. While many bacteria exhibited robust positive interactions, *Malassezia* uniquely showed negative correlations, particularly with *Lactobacillus* and *Lacticaseibacillus* genera. This data reaffirms *Malassezia*'s distinct ecological role, potentially as a dominant competitor or inhibitor. The observation that some of the most abundant bacterial genera, such as *Cutibacterium*, *Staphylococcus*, and *Corynebacterium,* were minimally interactive could reflect niche differentiation and a potential contribution to the stability of the microbial community.

Independently from the population dynamics, EUTOPLAC induced a significant increase in the relative abundance of *L. crispatus, L. paracasei,* and other *Lactobacillus* and *Lacticaseibacillus* species at T8. This suggests that applying the oily cream may provide favorable conditions for the probiotics in the EUTOPLAC formulation and other *Lactobacillus* and *Lacticaseibacillus* commensal species.

Surface adhesion and biofilm formation are essential for skin colonization^40^. Thus, we examined the in vitro adhesion and biofilm formation capacities of *L. crispatus* P17631 and *L. paracasei* I1688 under varying growth conditions. Compared to *L. paracasei* I1688, *L. crispatus* P17631 showed superior surface adhesion in aerobic conditions. However, under anaerobic conditions, their adhesion capacities were comparable.

Additionally, *L. paracasei* I1688 outpaced *L. crispatus* P17631 in biomass production under anaerobic conditions. The findings highlight how growth conditions can significantly influence bacterial proliferation, adhesion, and biomass production. They underscore the importance of considering these environmental factors when studying or exploiting these bacteria's behaviors and potential applications. Previous research has established that different strains of *L. crispatus* and *L. paracasei* are able to produce biofilms. Research has shown that *L. crispatus* forms biofilms in the vaginal environment, which aids in the competition against pathogenic *Candida* clinical isolates^41^. However, other studies note that not all *L. crispatus* strains of vaginal origin were able to form a biofilm^42,43^. These findings suggest a great strain-specific variability in biofilm production amongst *L. crispatus* isolated from the same environment. Similarly, *L. paracasei* ATCC334 has been shown to form biofilms in harsh conditions. This suggests that delivering probiotics in biofilm-like microcolonies could boost bacterial functionalities^44^. *L. paracasei*, which is known to produce molecules with antimicrobial and surfactant properties, is able to form biofilm and inhibit *Streptococcus oralis* growth in vitro^45^. In conjunction with other characteristics, the biofilm-forming ability makes *L. crispatus* and *L. paracasei* effective probiotics for skin health.

Biofilm interactions are pivotal in the cutaneous ecosystem, where they significantly influence microbial dynamics. The biofilm-forming abilities of *L. crispatus* P17631 and *L. paracasei* I1688 might play a crucial role in modulating the skin microbiome, particularly in their interactions with *Malassezia*. By producing substantial biomass, these probiotics could establish colonies that inhibit or outcompete *Malassezia* biofilms, potentially contributing to the therapeutic effects of EUTOPLAC in managing SD. Recent studies illustrate this antagonistic interplay, such as *Malassezia*'s secretion of proteases capable of disrupting *S. aureus* biofilms, delineating a competitive relationship between these skin inhabitants^46^. Furthermore, a contrasting interaction pattern between two *Staphylococcus* species and *M. restricta* has been observed. While *S. aureus* and *M. restricta* interactions are less pronounced, *S. epidermidis* exhibits a significant antagonistic response, actively upregulating defense and biofilm-related genes when in contact with *M. restricta* ^47^(Lyou et al., 2023).

Additionally, *Lactobacillus* species are known to secrete a variety of antifungal metabolites, such as organic acids and biosurfactants, which have been documented to inhibit *Candida* growth in the vaginal environment^48^. These findings suggest that *L. crispatus* P17631 and *L. paracasei* I1688 could similarly influence *Malassezia* populations, either directly through antifungal activity or indirectly by altering the local microenvironment.

Understanding the intricate biofilm-mediated interactions between probiotics, *Malassezia*, and other skin microbiota is fundamental in elucidating the pathogenesis of SD. Our study's insights into the mycobiome-bacteriome interplay following EUTOPLAC application underline the necessity for in-depth research to unravel the exact mechanisms behind these dynamic relationships and their implications for effective SD treatment strategies.

Although the insights gained in this study are promising, further research is needed to fully elucidate the potential benefits and mechanisms of action of EUTOPLAC in SD management and mycobiome-bacteriome modulation. Randomized controlled trials with larger sample sizes and longer-term follow-ups will be crucial in advancing our understanding and validating the use of probiotics-based products as a treatment strategy for SD.

Overall, our findings underscore that EUTOPLAC administration significantly reduces SD symptom severity, highlighting its therapeutic potential for SD. This outcome indicates the therapeutic potential of EUTOPLAC in SD treatment and points possible new probiotic-based treatment approaches. EUTOPLAC application led to transient, targeted changes in fungal diversity, particularly affecting the relative abundance of *Malassezia*. Distinct dynamics in bacterial communities were also evident, marked by increased probiotic genera and a substantial decline in *S. aureus*, which has been linked to SD. These findings highlight the potential of microbiome-modulating therapies for treating SD. However, understanding such interventions' temporal and species-specific effects requires extensive research.

## Methods

### Study design

This study was an open-label exploratory study in patients with SD conducted at the Department of Clinical Dermatology, San Gallicano Dermatological Institute, of Rome, Italy. This study was carried out in accordance with the Declaration of Helsinki and in agreement with the ethical guidelines of the European Independent Ethics Committee. The protocol was submitted and approved by the ethics committee with Prot. 8173 N1189/19. Informed consent was obtained from all subjects and/or their legal guardian(s) for publication of identifying information/images in an online open-access publication.

### Study subjects

#### Selection criteria of patients with SD

The study enrolled 25 adult patients diagnosed with moderate to severe Seborrheic Dermatitis (SD), all recruited from September 2020 to January, 2021. Patients were selected from individuals visiting the San Gallicano Dermatological Institute for the first time, while the control group was recruited from the Institute's healthcare staff. SD was determined based on the clinical manifestation of red, flaky, oily patches found on the scalp, nasolabial folds, ears, eyebrows, and chest, often accompanied by itching or hair loss. Two experienced dermatologists, with an interexaminer agreement of about 90%, evaluated the severity scores and clinical grading of SD. The Seborrheic Dermatitis Area and Severity Index (SDASI), with a score range of 0–12.6, assessed SD severity^49,50^. All the SD patients were treated with the EUTOPLAC cream, containing *L. crispatus* P17631 and *L. paracasei* I1688 in a ratio 29:1, not less than 10^6^ CFU/capsule, once a day (before nocturnal rest) on their nasolabial fold and glabella for one week. The SDASI scores were evaluated at the enrolment (T0), after seven days of treatment (T8), and three weeks after treatment (T28).

#### Exclusion criteria

All subjects with concurrent skin inflammatory disorders or signs of systemic illness; SD patients with previous topical antifungal, antibacterial, and/or steroid treatment for SD or dandruff within one month prior to sampling were excluded.

#### Sample collection

The samples were collected by dermatologists with commercially available sterile swabs (COPAN swabs, Brescia, Italy) from nasolabial fold and glabella from patients with SD and the skin of healthy subjects (HS) at the enrolment (T0), the day following the end of treatment (T8), and three weeks after treatment (T28). Swabs were brought to the Microbiology and Virology Laboratory of San Gallicano Institute and stored at − 80 °C until mycobiome-bacteriome analysis.

#### Mycobiome-bacteriome profiling and bioinformatics analysis

Extracted DNA was amplified by PCR with dual-index primers targeting the V1-V3 regions of the bacterial 16S rRNA gene and the internal transcribed spacer 2 (ITS2) to determine fungal composition. A sterile sample tube that had undergone the same DNA extraction and PCR amplification procedures was used for quality control. Before sequencing, amplicons were purified using the Agencourt® AMPure XP PCR purification system (Beckman Coulter, Milan, Italy), and equal amounts (10 nM) of the sample’s DNA were pooled and diluted to reach a 4-nM concentration. Finally, 5 pM of the denatured libraries were used to generate sequences using the 2 × 250 cycles MiSeq Reagent kit (Illumina, San Diego, CA) on an Illumina MiSeq instrument^51^.

The bioinformatics analysis of both 16S and ITS2 data was conducted employing QIIME2 software (v. 2022.08)^52^.

Differences in microbial communities were measured in terms of alpha and beta diversity after read-depth rarefaction. The Shannon and Pielou indexes were used to measure alpha diversity, with the Kruskal–Wallis tests assessing statistical significance. Beta diversity was evaluated using the Bray Curtis metric, with the resulting distance matrix visualized through Principal Coordinate Analysis (PCoA). The Permutational Multivariate Analysis of Variance (PERMANOVA) was used to assess significance. The taxonomic classification of 16S data was performed using the SILVA database (v.138)^53^, while for ITS2 classification, the UNITE database (v.8_05-2021)^54^ was chosen. The differential analysis of microbial abundance from longitudinal data at phylum and genus level across selected groups was performed using the Microbiome Multivariable Associations with Linear Models (MaAsLin 2) package (v. 1.14.1)^55^ setting inter-subject variation as a random effect to account for repeated measures.

### Co‐occurrence/co‐exclusion relationships network analysis

For each time point, a correlation network was computed independently. A first filtering step was performed by removing low OTU abundances with a total number of reads of less than 2000 across all samples or less than 4000 for the correlation network considering all times together, representing an average relative abundance of less than ~ 0.05% for each taxon. Subsequently, for each separate group, OTUs with a count of zero were filtered out, and the remaining unique entries were tested for their correlations using R version 4.3.1 function *cor* with Spearman’s statistic, which is used to estimate a rank-based measure of association. These are more robust and recommended if the data do not necessarily come from a bivariate normal distribution. The network was subsequently calculated and visualized by *qgraph*^56^ R package version 1.9.5. It used a cutoff threshold of 0.4 (or 0.5 for the bacteria and fungi network) for both positive and negative correlation coefficient values to remove less relevant relationships in the taxonomic network. The threshold to cut the scaling of edges in width and color saturation was set to 0.5 for both positive and negative correlation. Wilcoxon tests were used to show the difference between the distribution of Spearman’s *r* values of the three-time points considered.

#### Evaluation of the biofilm formation

Sterile 96-well polystyrene plates were inoculated with 200 μL of an initial bacterial suspension (10^7^ CFU/mL) in BHI broth incubated at 37 °C for 48 h in aerobic and anaerobic conditions without shaking. The GasPak anaerobic system created an oxygen-free environment (Becton Dickinson, Franklin Lakes, NJ, USA). As described previously, biofilm formation was assayed using crystal violet staining in 96-well microtiter plates^57^.

#### Biofilm Ring Test® (BRT)

Surface adhesion for *L. crispatus* P17631 and *L. paracasei* I1688 strains was analyzed using the BioFilm Ring Test (BRT) as previously described^58^, using the reagents and equipment provided by the Biofilm Ring Test kit (KITC004) and analyzed with BFC Elements 3.0 software (Biofilm Control, Saint Beauzire, France). The GasPak anaerobic system created an oxygen-free environment (Becton Dickinson, Franklin Lakes, NJ, USA). *S. aureus* strain ATCC 6538 and *Staphylococcus epidermidis* ATCC 12,228 were included in each plate as the standard reference and internal control, respectively. Each strain was analyzed in duplicate, and experiments were repeated three times.

#### Biofilm imaging

Colonies of *L. crispatus* P17631 and *L. paracasei* I1688, cultivated overnight on blood agar plates, were used to prepare a bacterial suspension. Specifically, the colonies were suspended in 3 ml of 0.45% saline solution (Air Life, Carefusion, CA, USA) until they reached a turbidity of 2.5 ± 0.3 on the McFarland scale, equating to roughly 1 × 10^8^ CFU/ml. This suspension was then diluted at a ratio of 1:1000 and transferred into 1 ml of BHI within μ-Slide, 8-well glass bottom chamber slides (Ibidi, Germany). The bacterial mixture was incubated at 37 °C over 48 h for biofilm development. After this period, the medium was discarded, and the samples were rinsed with 0.45% saline solution. Biofilm cells were then stained using the LIVE/DEAD BacLight kit (Life Technologies, New York, NY, USA), following the manufacturer's guidelines, and examined with an Apotome system (Zeiss, Oberkochen, Germany) connected to an Axio Observer inverted fluorescence microscope (Zeiss). Data were analyzed with the ZEN 3.2 (blue edition) software (Zeiss).

#### Statistical analysis

Data are presented as mean ± standard error of the mean. Statistical analyses were conducted using ANOVA with Tukey post hoc, Friedman and post hoc Nemenyi test, or Kruskal–Wallis with Dunn’s post hoc tests, followed by suitable p-value correction. For Beta diversity, PERMANOVA was applied to Bray–Curtis distance matrices. A p-value of 0.05 or less was deemed statistically significant. All statistical analyses were performed using IBM SPSS v.21 statistics software (IBM, Chicago, IL, USA).

### Supplementary Information

Supplementary Information.

## Data Availability

The authors declare that the data supporting the findings of this study are available within the paper or from the corresponding author upon reasonable request. The data for this study have been deposited in the European Nucleotide Archive (ENA) at EMBL-EBI under accession number PRJEB66726 (https://www.ebi.ac.uk/ena/browser/view/PRJEB66726).
