# Supplementary material for: Modulating the skin mycobiome-bacteriome and treating seborrheic dermatitis with a probiotic-enriched oily suspension
Source: Sci Rep. 2024 Feb 1;14:2722. doi: 10.1038/s41598-024-53016-0 (PMC10834955; doi:10.1038/s41598-024-53016-0)
Supplement: Supplementary file 1 — Supplementary Information. [file 41598_2024_53016_MOESM1_ESM.pdf]

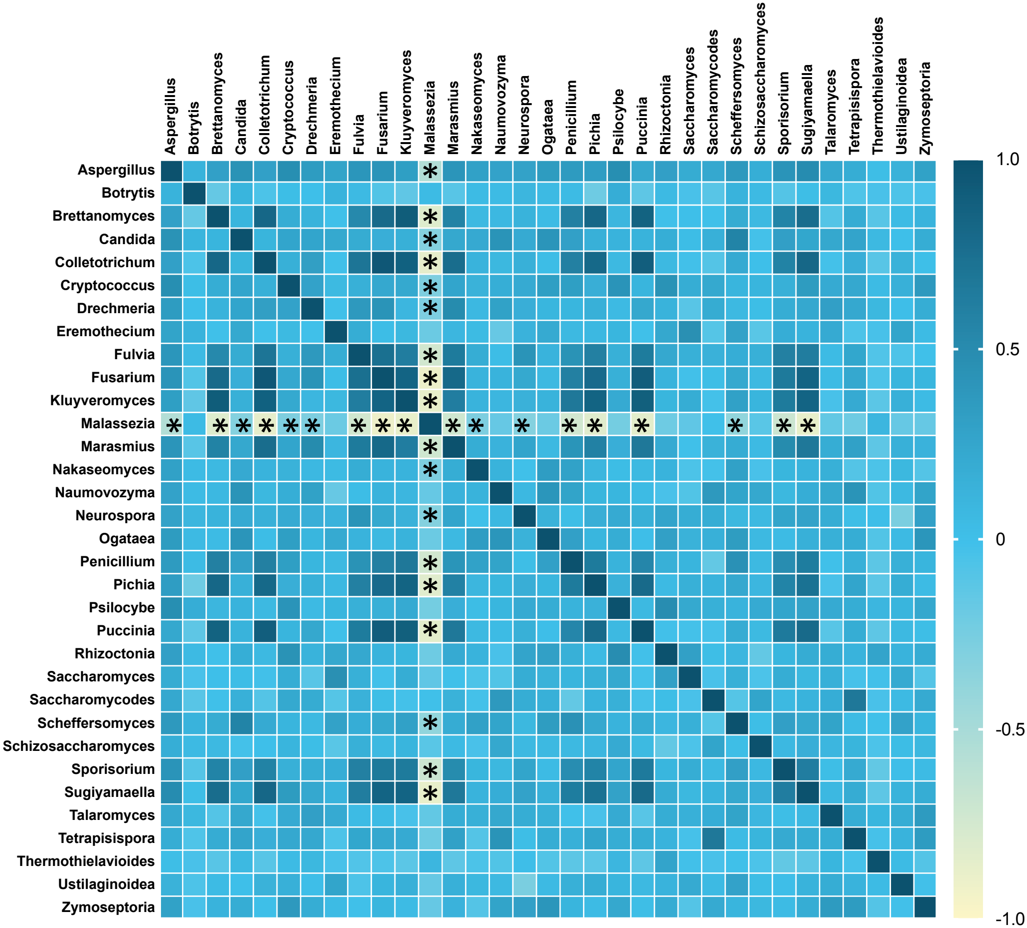

Supplementary Figure 1. Color coded correlation matrix visualizing significant correlations (* $p < 0.05$) between fungal genera relative abundance data.
